# Abiotic and biotic factors controlling the dynamics of soil respiration in a coastal dune ecosystem in western Japan

**DOI:** 10.1038/s41598-022-17787-8

**Published:** 2022-08-22

**Authors:** Munemasa Teramoto, Toru Hamamoto, Naishen Liang, Takeshi Taniguchi, Takehiko Y. Ito, Richa Hu, Norikazu Yamanaka

**Affiliations:** 1 Arid Land Research Center, Tottori University, Hamasaka, Tottori 680-0001 Japan; 2 Graduate School of Agricultural Science, Tohoku University, Sendai, Miyagi 980-8572 Japan; 3 Earth System Division, National Institute for Environmental Studies, Tsukuba, Ibaraki 305-8506 Japan; 4 International Platform for Dryland Research and Education, Tottori University, Hamasaka, Tottori, 680-0001 Japan; 5 The United Graduate School of Agricultural Sciences, Tottori University, Koyama-Minami, Tottori, 680-8553 Japan

**Keywords:** Ecosystem ecology, Ecosystem services, Ecology, Ecology, Environmental sciences

## Abstract

In this study, we examined the abiotic and biotic factors controlling the dynamics of soil respiration (*R*_s_) while considering the zonal distribution of plant species in a coastal dune ecosystem in western Japan, based on periodic *R*_s_ data and continuous environmental data. We set four measurement plots with different vegetation compositions: plot 1 on bare sand; plot 2 on a cluster of young *Vitex rotundifolia* seedlings; plot 3 on a mixture of *Artemisia capillaris* and *V. rotundifolia*; and plot 4 on the inland boundary between the coastal vegetation zone and a *Pinus thunbergii* forest. *R*_s_ increased exponentially along with the seasonal rise in soil temperature, but summer drought stress markedly decreased *R*_s_ in plots 3 and 4. There was a significant positive correlation between the natural logarithm of belowground plant biomass and *R*_s_ in autumn. Our findings indicate that the seasonal dynamics of *R*_s_ in this coastal dune ecosystem are controlled by abiotic factors (soil temperature and soil moisture), but the response of *R*_s_ to drought stress in summer varied among plots that differed in dominant vegetation species. Our findings also indicated that the spatial dynamics of *R*_s_ are mainly controlled by the distribution of belowground plant biomass and autotrophic respiration.

## Introduction

Coastal dunes are important ecosystems that are inhabited by unique vegetation communities, but they are being threatened by the recent climate change and developmental pressures^1^. On the other hand, coastal dune ecosystems are gaining attention as green infrastructure that can provide many kinds of ecosystem services^2^, including carbon sequestration and storage^3^. A soil carbon analysis by Drius et al.^4^ suggested that Italian coastal dunes in the Natura 2000 network are carbon sinks. We have limited information, however, on the carbon cycle in coastal dune ecosystems based on CO_2_ flux observation data. Therefore, more CO_2_ flux research in coastal dunes is needed to clarify the value of coastal dune ecosystems from the viewpoint of climate change mitigation. This new information may also contribute to protecting dune ecosystems and establishing sustainable management strategies for these vulnerable ecosystems.

Soil respiration (*R*_s_) is the second-largest carbon flux in terrestrial ecosystems and a major component of the global carbon cycle^5^. *R*_s_ consists of heterotrophic respiration (*R*_h_, decomposition of plant litter and soil organic carbon (SOC) by microbiota) and autotrophic respiration (*R*_a_) that comes from plant roots, mycorrhiza, and other rhizosphere microorganisms^6^. Although several studies have estimated global *R*_s_^5,7^, large uncertainty in the estimation still remains^8^ and may be partly explained by spatial bias for *R*_s_^9^. Not only spatial bias but also bias associated with ecosystem types might contribute to the uncertainty. For example, according to the recent database of *R*_s_ by Jian et al.^10^, *R*_s_ data in forest ecosystems (especially in temperate regions) is the most frequently recorded category from the viewpoint of the number of data in the database. More research in a wider array of ecosystems, however, would help improve *R*_s_ estimation as well as our understanding of the mechanism underlying the *R*_s_ response to environmental factors.

In most ecosystems, abiotic factors such as soil temperature and soil moisture are the major environmental factors controlling the dynamics of *R*_s_, but the magnitude and the direction (increase or decrease) of the *R*_s_ response to those factors vary among ecosystems. Generally, *R*_s_ increases exponentially along with a rise in soil temperature^11–13^, whereas *R*_s_ has a “mountain-shaped” relationship with soil moisture, because conditions that are too dry or too wet suppress *R*_s_^14^. Gao et al.^15^ observed an exponential relationship between soil temperature and *R*_s_ in plantation forests and a secondary forest in coastal dunes, as typically observed in other terrestrial ecosystems; they also reported that soil moisture was positively related to *R*_s_ in several stands among the study sites. Their findings suggest that soil temperature and soil moisture also exert strong control over the seasonal dynamics of *R*_s_ in coastal dunes.

In addition to abiotic factors, biotic factors are also reported to regulate the dynamics of *R*_s_ in coastal dune ecosystems. Previous studies suggested that biotic factors like root biomass and microbial population were also important indicators for the dynamics of *R*_s_ in coastal dune ecosystems^15,16^, however, it needs more discussion to get a consensus about which biotic factor is the most dominant one. Chapman^17^ estimated that 70% of *R*_s_ is caused by root respiration, based on surveys in several heathland ecosystems that included dune-heath ecosystems, suggesting a strong influence of vegetation on *R*_s_ in those ecosystems. Because coastal dune ecosystems are generally carbon-limited, interactions between vegetation and *R*_s_ might be more clearly observed than in other ecosystems.

In coastal dune ecosystems, a zonal distribution (zonation) of coastal plant species with distance from the shoreline is typically observed^18^. As the distance from the shoreline increases, the vegetation gradually changes from herbs to shrubs and tree species^4,19^, and the amount of soil organic matter is also positively correlated with the distance^20^. Because plant communities can be a strong driver of *R*_s_^21^, the dynamics of *R*_s_ and the response to environmental factors may differ between vegetation zones. Thus, for a better understanding of *R*_s_ dynamics and the mechanism underlying the *R*_s_ response to abiotic and biotic factors in coastal dune ecosystems, it is necessary to account for the zonal distribution of plant communities. No study has yet examined, however, if the *R*_s_ dynamics and responses to abiotic and biotic factors differ among plots dominated by different coastal plant species.

In this study, we investigated the dynamics of *R*_s_ in a Japanese coastal dune ecosystem focusing on abiotic factors (soil temperature and soil moisture) and biotic factors (belowground plant biomass and microbial abundance), while considering the difference in dominant coastal vegetation. We hypothesized that the relationships between the controlling factors (abiotic and biotic) and *R*_s_ vary among plots dominated by different vegetation species. We also aimed to identify the mechanism(s) that caused the difference in the response of *R*_s_ using ecological and microbial analysis.

## Results

### Environmental data

**Figure 1:**
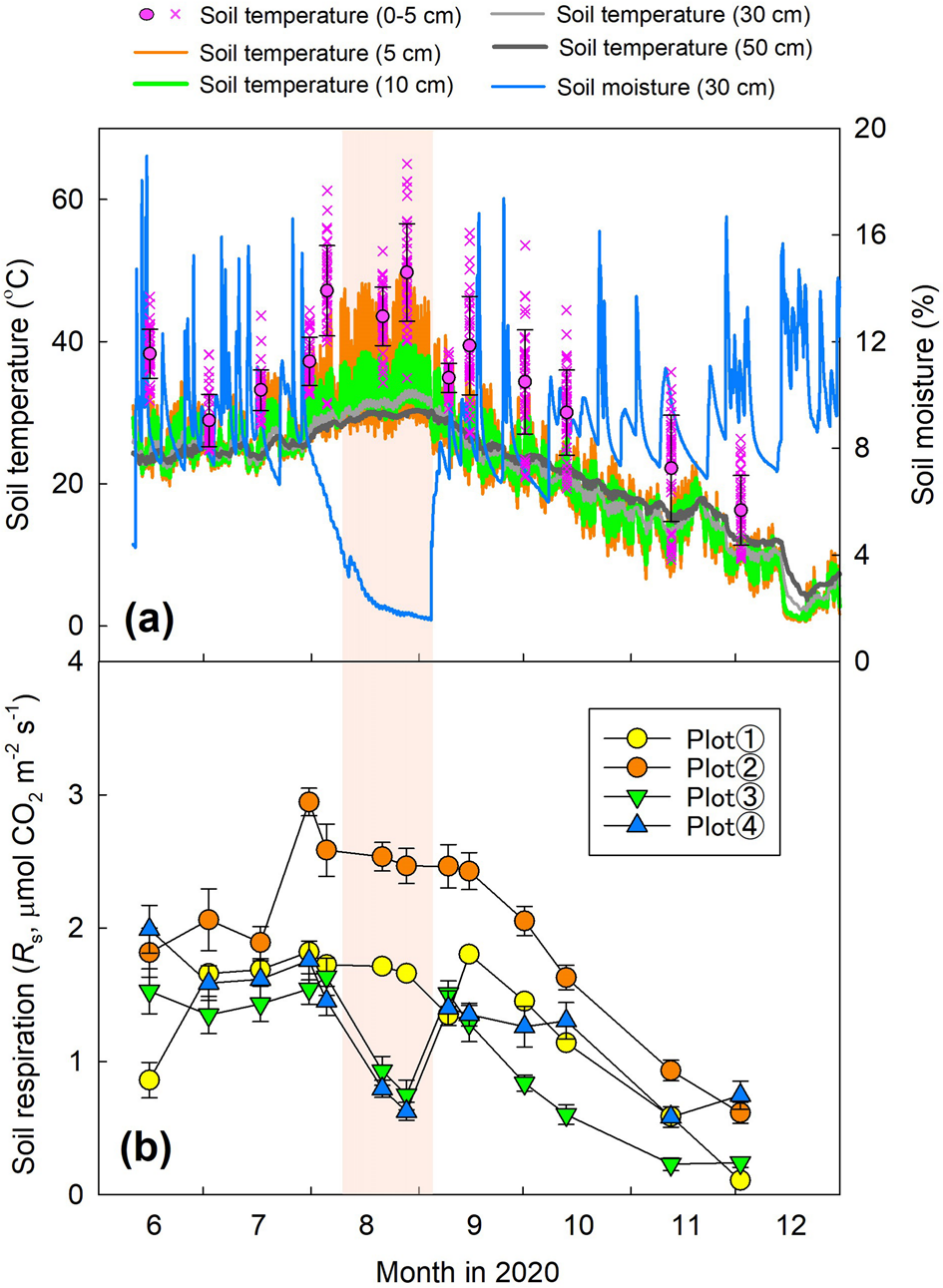
(**a**) Seasonal dynamics of soil moisture at a depth of 30 cm (averaged value of CS616 sensors in plots 1, 3, and 4) and soil temperature at depths of 0–5, 5, 10, 30, and 50 cm; (**b**) seasonal dynamics of *R*_s_ in each measurement plot. The soil temperature at the depth of 0–5 cm for all 40 measurement points in plots 1–4 is shown by pink crosses, and the average value (mean ± SD) during the daytime is shown by pink circles. Soil temperature at depths of 5, 10, and 30 cm is the average value of reference soil temperature (thermocouple) at the center of plot 3 and stand-alone soil temperature sensors in plots 1 and 4. Soil temperature at a depth of 50 cm is the reference soil temperature at the center of plot 3. Bars in (**b**) show the standard error of the mean (*n* = 10). The area of light orange represents the drought period (10 August to 4 September) when the daily averaged soil moisture was less than 3.9%, the threshold soil moisture value. This figure was created using Sigmaplot 14.5 software (Systat Software, San Jose, CA, USA, https://systatsoftware.com/sigmaplot/).

The time series of the soil moisture at a depth of 30 cm and soil temperature at depths of 0–5, 5, 10, 30, and 50 cm are shown in Fig. 1a. The maximum soil temperature from June to December 2020 at 0–5 cm, based on data collected at each measurement point during each *R*_s_ measurement, was 65.0 °C measured during the daytime in late August. The maximum soil temperatures based on the average of 30-min continuous measurements at depths of 5, 10, 30, and 50 cm were 49.7, 40.1, 33.2, and 30.4 °C, respectively, in late August. The minimum soil temperature from June to December 2020 at 0–5 cm during *R*_s_ measurement was 9.3 °C during the daytime in mid-November. The minimum soil temperature at depths of 5, 10, 30, and 50 cm was 0.6, 0.9, 2.2, and 3.7 °C, respectively, in late December.

The maximum average soil moisture at the depth of 30 cm was 19.0% in the middle of June, the early summer rainy season, and the minimum value was 1.6% in early September (Fig. 1a). The remarkable decrease in soil moisture was due to limited precipitation in August. The monthly precipitation recorded at the meteorological observatory in Tottori (Japan Meteorological Agency) in August was 8.5 mm, the lowest monthly precipitation in August in the last 50 years.

### Dynamics of *R*_s_ in each plot

*R*_s_ values in plots 1–3 peaked at the end of July to early August, whereas *R*_s_ in plot 4 peaked in the middle of June (Fig. 1b). The maximum *R*_s_ values were 1.82 ± 0.05, 2.95 ± 0.10, 1.63 ± 0.14, and 1.99 ± 0.18 μmol CO_2_ m^−2^ s^−1^ in plots 1 to 4, respectively (mean ± SE, *n* = 10). Minimum *R*_s_ values in each plot were observed from mid-November to early December and were 0.11 ± 0.004, 0.61 ± 0.08, 0.23 ± 0.05, and 0.58 ± 0.08 μmol CO_2_ m^−2^ s^−1^ in plots 1–4, respectively (mean ± SE, *n* = 10). In plots 3 and 4, *R*_s_ was also markedly decreased at the end of August. During the measurement period from 15 June to 2 December (13 *R*_s_ measurements), the average *R*_s_ value in each plot was 1.35 ± 0.53, 2.03 ± 0.67, 1.07 ± 0.50, and 1.27 ± 0.45 μmol CO_2_ m^−2^ s^−1^, respectively (mean ± SD).

### Soil temperature and soil moisture response of ***R***_s_

**Figure 2:**
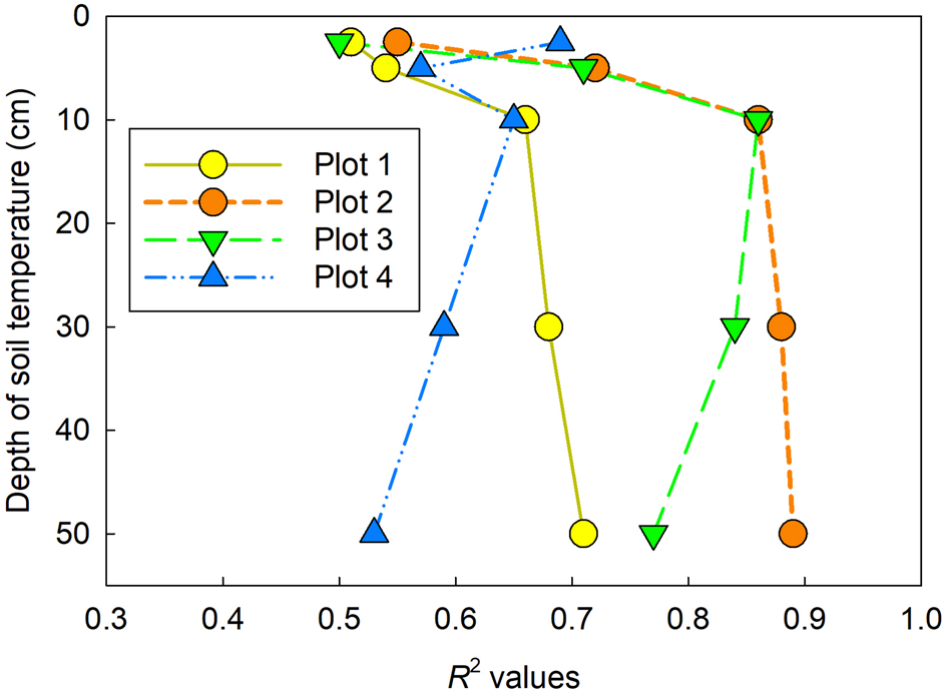
Change of *R*^2^ values based on the relationships between soil temperature at each depth and average *R*_s_ in each plot. For this analysis, data for late August were removed to avoid the period of drought stress. Data were measured continuously using an environmental measurement system and standalone soil temperature sensors near each plot at depths of 5, 10, and 30 cm. Soil temperature at the depth of 0–5 cm was measured simultaneously with *R*_s_ at each measurement point. Soil temperature at a depth of 50 cm was the reference soil temperature at the center of plot 3. This figure was created using Sigmaplot 14.5 software (Systat Software, San Jose, CA, USA, https://systatsoftware.com/sigmaplot/).

**Figure 3:**
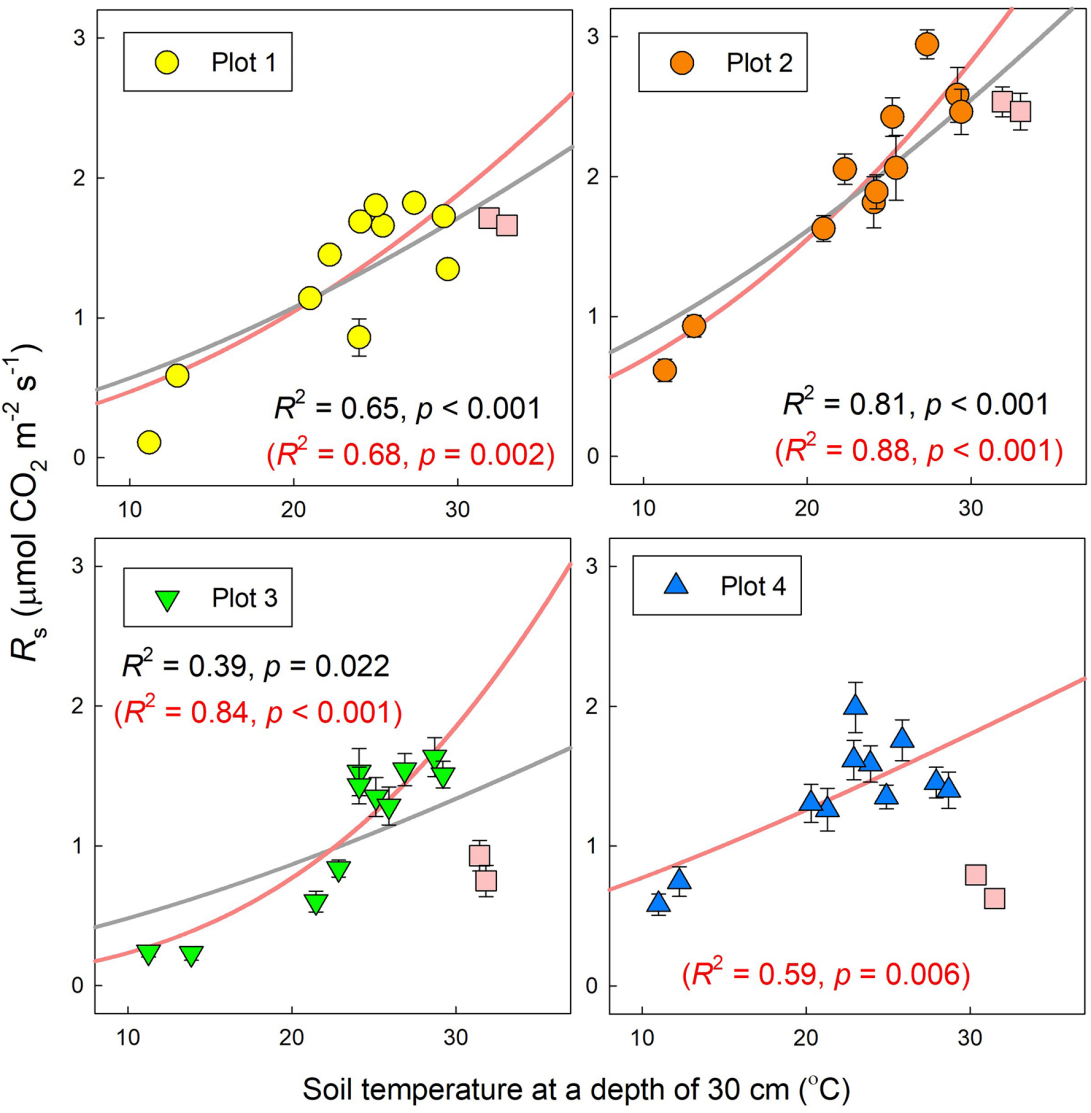
Relationships between soil temperature at 30 cm depth and averaged *R*_s_ in each plot. Pink squares represent data in late August 2020. Gray lines with black *R*^2^ and *p* values, show regression lines obtained by fitting Eq. (3) to all data ($R_{{\text{s}}} = R_{{{\text{ref}}}} {\text{e}}^{{E_{0} \times \left( {\frac{1}{{T_{{{\text{ref}}}} - T_{0} }} - \frac{1}{{T_{{\text{s}}} - T_{0} }}} \right)}}$). Red lines with red *R*^2^ and *p* values, are those obtained by excluding data from late August during the drought period. Bars show the standard error of the mean (*n* = 10). This figure was created using Sigmaplot 14.5 software (Systat Software, San Jose, CA, USA, https://systatsoftware.com/sigmaplot/).

*R*_s_ increased exponentially along with the seasonal rise in soil temperature, except for late August, and the goodness of fit (*R*^2^) was best at a depth of 50, 50, 10, and 0–5 cm in plots 1–4, respectively (Fig. 2). In this study, we selected the soil temperature at the depth of 30 cm as the standard for analysis of *R*_s_. Significant exponential relationships between 30-cm soil temperature and *R*_s_ were observed in plots 1, 2, and 3 when we used all data (Fig. 3, *p* < 0.001 in plots 1 and 2, *p* = 0.022 in plot 3), whereas there was no significant exponential relationship between soil temperature and *R*_s_ in plot 4 (*p* = 0.344). However, if we excluded data collected in late August (21 and 28 August), the drought period, a significant exponential relationship was also confirmed in plot 4 (Fig. 3, *p* = 0.006).

**Figure 4:**
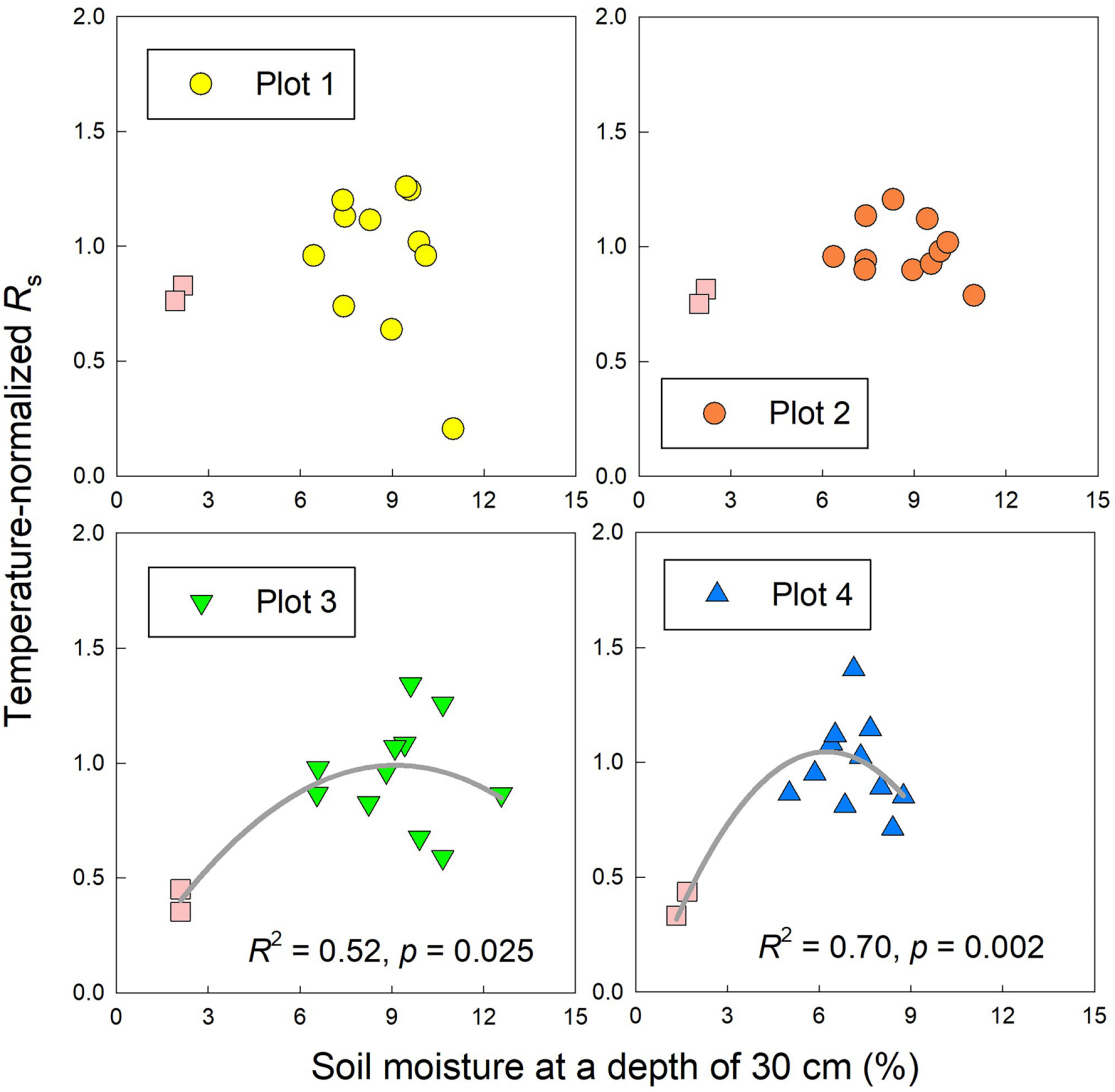
Relationships between soil moisture at a depth of 30 cm and temperature-normalized soil respiration (*R*_sN_, observed *R*_s_/modeled *R*_s_) in each measurement plot. Gray lines show significant (*p* < 0.05) relationships between soil moisture and *R*_sN_ fitted with Eq. (4) ($R_{{{\text{sN}}}} = c_{1} {\uptheta }^{2} + c_{2} {{\uptheta + }}c_{3} (c_{1} < 0)$). Pink squares represent *R*_sN_ during the drought period. This figure was created using Sigmaplot 14.5 software (Systat Software, San Jose, CA, USA, https://systatsoftware.com/sigmaplot/).

Using the soil temperature response curve of *R*_s_ for which data in late August (drought period) were excluded, we analyzed the relationships between soil moisture at a depth of 30 cm and temperature-normalized *R*_s_ (*R*_sN_) in each plot. These relationships were significant in plot 3 (*p* = 0.025) and plot 4 (*p* = 0.002, Fig. 4), but they were not significant in plot 1 (*p* = 0.232) or plot 2 (*p* = 0.055). Ranges of *R*_sN_ during the drought period from plots 1 to 4 were 0.76–0.83, 0.75–0.82, 0.35–0.45, and 0.33–0.44, respectively (Fig. 4).

### Belowground plant biomass

**Figure 5:**
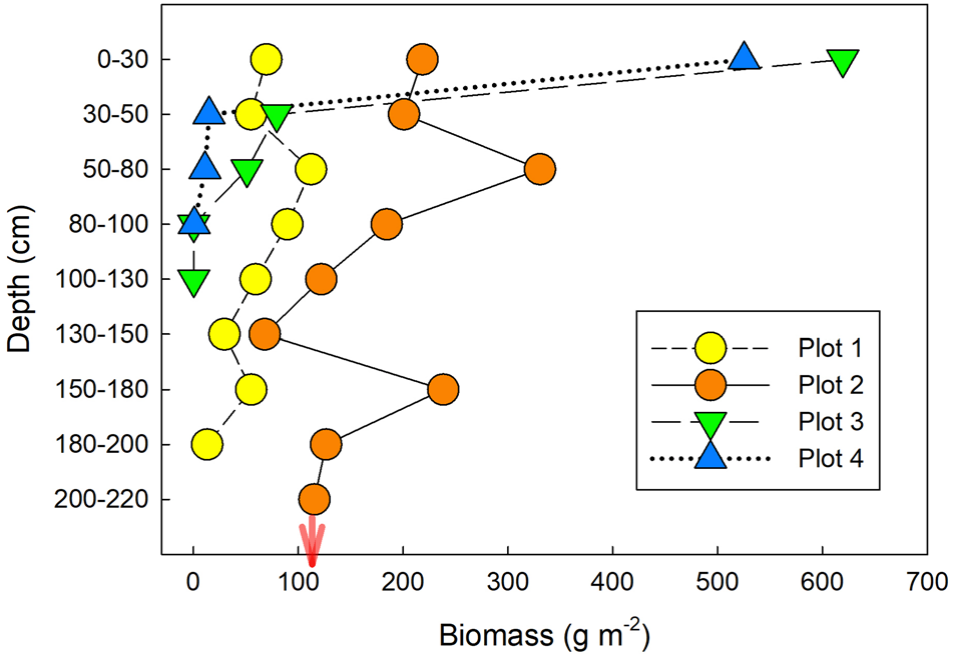
Profile of belowground plant biomass in each measurement plot to depths of 100–220 cm from 18 May to 8 June 2021 (*n* = 1). The red arrow indicates that roots were distributed below 220-cm depth. This figure was created using Sigmaplot 14.5 software (Systat Software, San Jose, CA, USA, https://systatsoftware.com/sigmaplot/).

We observed a large variation in the distribution of total belowground plant biomass (BPB) and rooting depth in each plot (Fig. 5). Total BPB to a depth of 220 cm was 483.8, 1604.6, 751.2, and 552.3 g m^−2^ for plots 1–4, respectively (*n* = 1). The majority of BPB was concentrated in the upper 30 cm in plot 3 (82%) and plot 4 (95%), and the BPB ratios to a depth of 50 cm were 93% (plot 3) and 98% (plot 4), respectively. In contrast, the BPB ratios in the upper 30 cm in plots 1 and 2 were both 14%, and the BPB ratios to a depth of 50 cm were both 26%.

### Influence of BPB on ***R***_s_

**Figure 6:**
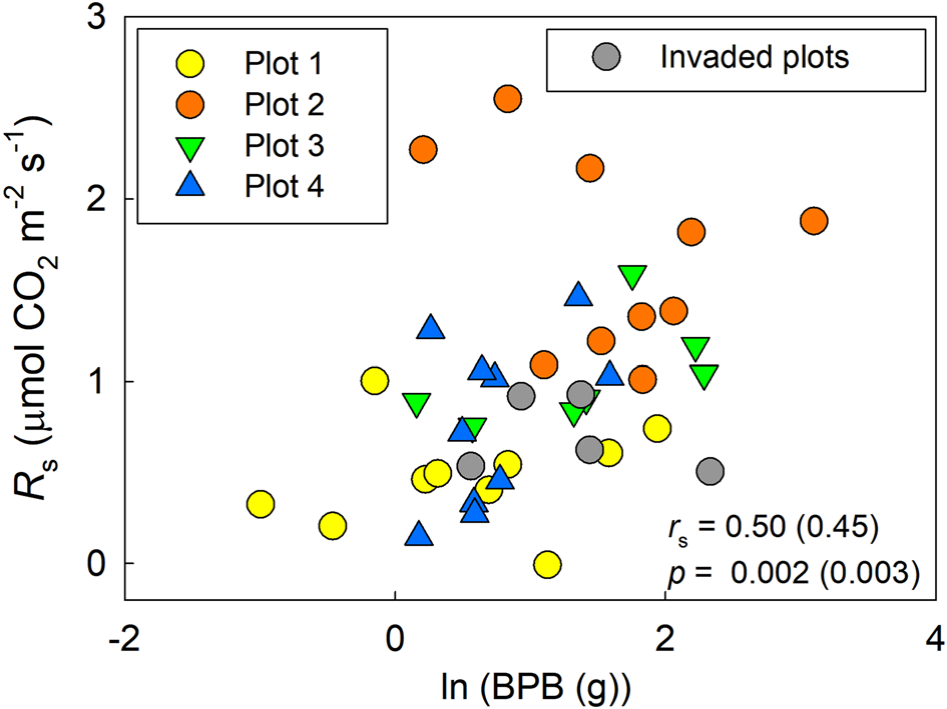
Relationship between the natural logarithm of belowground plant biomass (BPB, g) to a depth of 50 cm in each subplot and *R*_s_ on 3 November before trench treatment. Values in the figure are Spearman’s rank correlation coefficient (*r*_s_) and *p* value; values in parentheses are those when we included invaded plots (gray circles) in the analysis. This figure was created using Sigmaplot 14.5 software (Systat Software, San Jose, CA, USA, https://systatsoftware.com/sigmaplot/).

There was no significant correlation between the natural logarithm of BPB in subplots and *R*_s_ on 3 November (1 day before trench treatment) when we analyzed separately in plot 1 (*p* = 0.327), plot 2 (*p* = 0.365), and plot 4 (*p* = 0.199), but the relationship was significant in plot 3 (Spearman’s rank correlation coefficient = 0.75, *p* = 0.038). However, there was a significant positive correlation (*p* = 0.002–0.003) when data in all subplots were analyzed together (Fig. 6).

**Figure 7:**
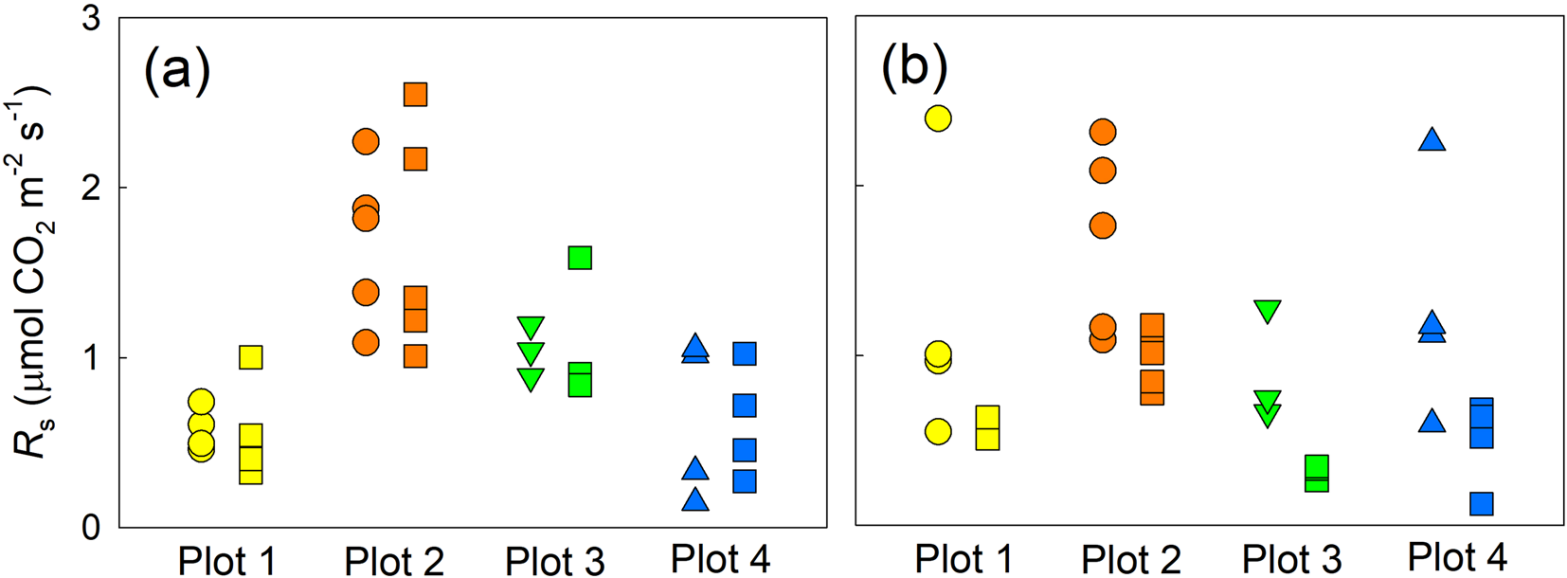
Comparison of *R*_s_ values between control and trenched plots (**a**) on 3 November before trench treatment and (**b**) on 19 November, 15 days after trench treatment (*n* = 4, 5, 3, and 4 for plots 1–4, respectively). *R*_s_ data in pre-trenched and trenched plots are shown as squares in all plots. Circles and triangles are *R*_s_ data in control plots. This figure was created using Sigmaplot 14.5 software (Systat Software, San Jose, CA, USA, https://systatsoftware.com/sigmaplot/).

Average *R*_s_ values in control and pre-trenched plots on 3 November 2020 were 0.58 ± 0.06 and 0.57 ± 0.15 μmol CO_2_ m^−2^ s^−1^ in plot 1, 1.69 ± 0.21 and 1.66 ± 0.30 μmol CO_2_ m^−2^ s^−1^ in plot 2, 1.04 ± 0.09 and 1.11 ± 0.24 μmol CO_2_ m^−2^ s^−1^ in plot 3, and 0.64 ± 0.23 and 0.62 ± 0.16 μmol CO_2_ m^−2^ s^−1^ in plot 4, respectively (mean ± SE, n = 4, 5, 3, and 4 for plots 1–4, Fig. 7a). Average soil CO_2_ efflux (*F*_c_) values measured in control and trenched plots 15 days after trench treatment on 19 November were 1.23 ± 0.40 and 0.58 ± 0.03 μmol CO_2_ m^−2^ s^−1^ in plot 1, 1.69 ± 0.24 and 0.98 ± 0.07 μmol CO_2_ m^−2^ s^−1^ in plot 2, 0.90 ± 0.19 and 0.31 ± 0.03 μmol CO_2_ m^−2^ s^−1^ in plot 3, and 1.29 ± 0.35 and 0.49 ± 0.13 μmol CO_2_ m^−2^ s^−1^ in plot 4, respectively (mean ± SE, n = 4, 5, 3, and 4 for plots 1–4, Fig. 7b). Based on the *F*_c_ data collected on 19 November, *R*_a_50_/*R*_s_ was calculated as 0.53, 0.42, 0.65, and 0.62 in plots 1–4, respectively.

### Soil organic carbon and microbial abundance

Total SOC stocks from 0 to 30 cm depth in plots 1–4 were 44.5 ± 1.5, 57.9 ± 3.6, 248.5 ± 30.5, and 328.2 ± 73.2 g C m^−2^ (mean ± SE, *n* = 3), respectively. The ratio of SOC stock in the upper 10 cm against SOC stock to a depth of 30 cm in plots 1 to 4 were 31%, 26%, 48%, and 61%, respectively. Total nitrogen stocks at 0 to 30 cm depth in plots 1–4 were 7.8 ± 0.7, 7.1 ± 0.3, 14.3 ± 1.1, and 13.5 ± 1.6 g N m^−2^ (mean ± SE, *n* = 3), respectively.

**Figure 8:**
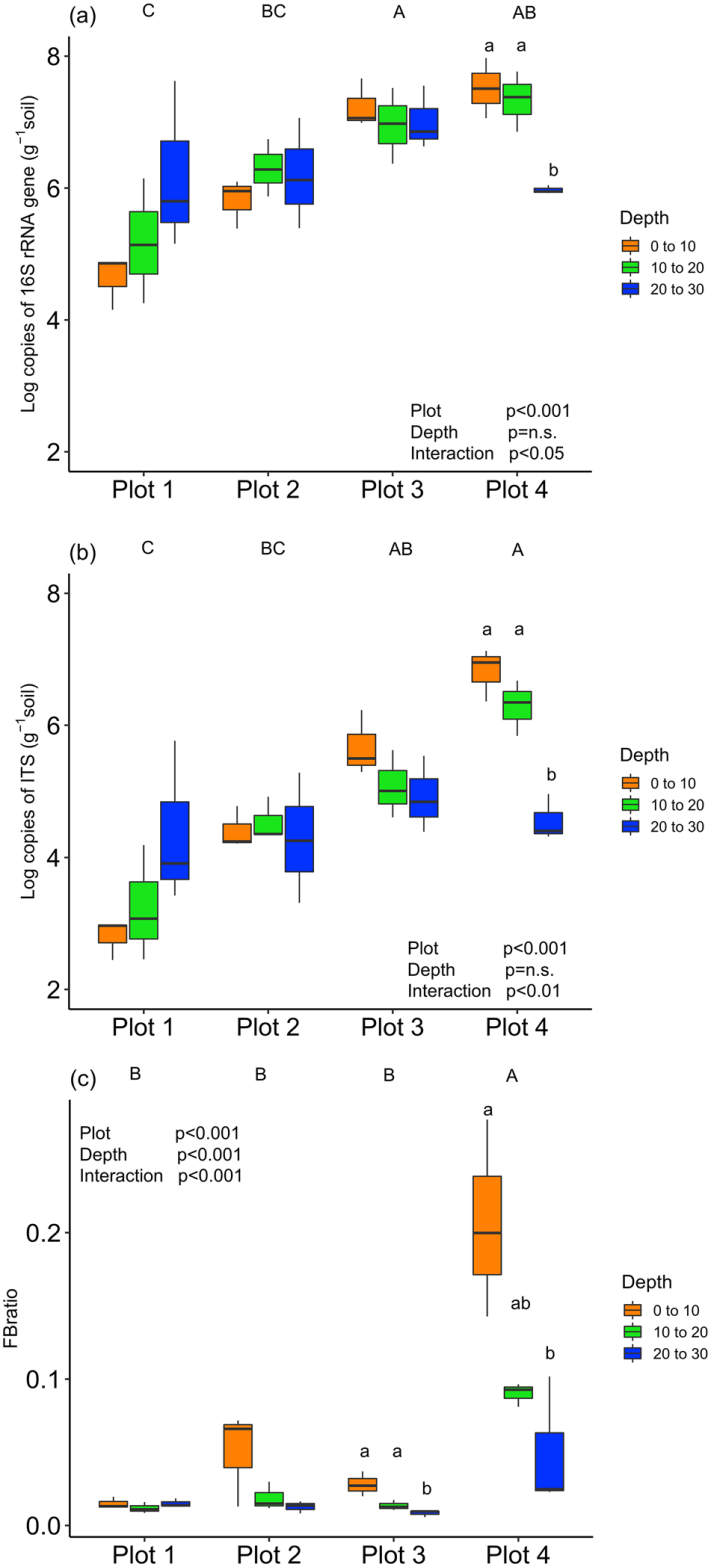
Gene abundances of (**a**) bacteria and archaea, (**b**) fungi, and (**c**) the ratio of fungi and bacteria. The level of significance was determined by two-way ANOVA. Uppercase letters indicate a statistically significant difference between vegetation plots when we averaged the values across soil depth (0–30 cm) and used the Tukey–Kramer test among vegetation plots. Lowercase letters indicate a significant difference between soil depths in each plot.

When averaged across soil depths, plots 3 and 4 had higher bacterial and fungal abundance than the other plots, whereas the bare plot (plot 1) showed the lowest bacterial and fungal abundance (Fig. 8a,b). Soil depth did not have significant effects on either bacterial or fungal abundance, but there were significant interactions between location and soil depth. Deeper soils (20–30 cm) only in plot 4 showed significantly lower microbial abundance when compared to the surface layer. Fungal abundances were from 5 to 125 times lower than bacterial abundances (Fig. 8c). There were significantly positive relationships between SOC stock (g C m^−2^) and log copies of genes for both bacteria (*R*^2^ = 0.39, *p* < 0.001) and fungi (*R*^2^ = 0.55, *p* < 0.001).

## Discussion

Our study showed that seasonal dynamics of *R*_s_ in a coastal dune ecosystem were controlled mainly by soil temperature, except for the dry summer period in August 2020. Generally, the soil temperature is the primary factor controlling *R*_s_ in many natural ecosystems^12,22,23^. Gao et al.^15^ also reported a strong relationship between soil temperature and *R*_s_ in subtropical coastal dunes in China. In most cases, soil temperature at shallower layers (e.g., 5 cm) is the appropriate parameter to explain the dynamics of *R*_s_ using exponential regressions^24–26^, because the litter layer and shallow layers of soil (e.g., A horizon), which contain more SOC than the deeper layer, contributes a large portion of the total *R*_s_^27–29^. Pavelka et al.^30^ showed that *R*^2^ values based on an exponential relationship between soil temperature and *R*_s_ decreased significantly at greater soil temperature depths in a Norway spruce forest and a mountain grassland in the Czech Republic. However, in plots 1 and 2 in our study, soil temperature at depths of 30 and 50 cm was a better indicator (the best fit soil temperature-depth) than that at 0–5 and 5 cm for seasonal dynamics of *R*_s_ (Fig. 2). Our result implies that the difference in best-fit soil temperature depth reflects the vertical distribution of the source of *R*_s_ along across soil depth. At least in plots 1 and 2, both the upper layer (above 30 cm) as well as the deeper layer (below 30 cm) appeared to contribute to *R*_s_ because BPB was distributed at depths of 200 cm and more (Fig. 5). In plots 3 and 4, on the other hand, 82–95% of BPB was concentrated within the top 30 cm. In addition, 48–61% of SOC was concentrated in the upper 10 cm in plots 3 and 4. Furthermore, the abundances of bacteria and fungi were both significantly higher in the upper 20 cm than at 30-cm depth in plot 4 (Fig. 8). These findings indicate that the shallower soil layer (above 30-cm depth) made a greater contribution to *R*_s_ in plots 3 and 4 as compared to that in the other plots, which may explain why soil temperature in the shallower layers (0–5 and 10 cm) fit the soil temperature response model of *R*_s_ well in plots 3 and 4. Most previous studies used surface soil temperature (0–10 cm) to examine the soil temperature response of *R*_s_—even in desert ecosystems where extreme surface soil temperatures of > 50 °C were observed^31^. Therefore, when performing temperature response analysis of *R*_s_, we recommend measuring soil temperature at multiple depths, including those at 30 cm and deeper in places where extreme soil temperature variation is expected, such as coastal dunes and deserts.

Soil moisture also had a strong impact on *R*_s_ especially in the drought period in August 2020, when a lack of precipitation contributed to drought stress and decreased soil moisture. Coastal dune ecosystems are easily influenced by drought stress^32,33^ because of the low water-holding capacity of sand^34^. Under drought stress, *R*_s_ is suppressed because of the limited microbial and plant activity, and *R*_h_ is expected to be more sensitive to drought stress than *R*_a_^35^. In our study, *R*_s_ markedly decreased in plots 3 and 4 under drought stress. There are few reports regarding the effect of drought stress on *R*_s_ in the coastal dune ecosystem, but our result for the soil moisture response of *R*_s_ (a mountain-shaped relationship) is consistent with previous studies in several other types of ecosystems^13,28^. Compared with plots 3 and 4, however, *R*_s_ was not remarkably decreased in plots 1 and 2 (Fig. 1b). The variety of rooting depths in each plot provides a clue about the difference. BPB in plots 1 (200-cm depth) and plot 2 (> 220 cm) was distributed more deeply than in plots 3 and 4, where more than 90% of BPB was in the upper 50 cm (Fig. 5). Therefore, the deeper rooting depth may have contributed to continued plant activity despite the drought stress at the surface and prevented a decrease of *R*_a_ in plots 1 and 2. Our findings indicate that the soil moisture response of *R*_s_ differed among vegetation zones of the coastal dune ecosystem, and drought stress markedly decreased *R*_s_ in some plots. Data from multiple years will be needed to confirm whether such drought stress occurs frequently in this coastal dune ecosystem because the precipitation in August 2020 was unusually limited compared with that in other years.

The significant positive relationship between the natural logarithm of BPB to 50-cm depth and *R*_s_ in November in our study suggests that the distribution of BPB is one factor controlling the spatial dynamics of *R*_s_ in the coastal dune ecosystem (Fig. 6). Positive linear relationships between root biomass and *R*_s_ have been reported^36–38^, whereas our result showed an logarithmic relationship between BPB and *R*_s_. This logarithmic relationship is reasonable when we consider that root respiration exponentially decreases with the increase of root diameter^17,39^. Lee^40^ reported an logarithmic relationship between root biomass and *R*_s_ using field-grown maple seedlings in central Korea. Our findings suggest that such an logarithmic relationship between root biomass and *R*_s_ is also applicable to coastal dune ecosystems.

The *R*_a_/*R*_s_ to a depth of 50 cm was estimated as 0.42–0.65 in November (Fig. 7). Although information about the *R*_a_/*R*_s_ is limited in coastal dune ecosystems, a study by Chapman^17^ in nine heathlands, including three dune-heath ecosystems, can serve as a reference. Chapman estimated that root respiration contributed up to 70% of *R*_s_ in heathland ecosystems, which is comparable to our result, suggesting that *R*_a_ is a large component of *R*_s_ in the SOC-limited dune ecosystem. Together, these results suggest that the distribution of BPB and the resulting *R*_a_ are major contributors to the spatial dynamics of *R*_s_ in coastal dune ecosystems.

In addition to plant roots, the mycorrhizal fungal network also contributes to *R*_s_. For example, Hogberg et al.^41^ reported a 50% decrease of *R*_s_ as a result of large-scale forest girdling, and they suggested the decrease was due to the inhibition of carbon translocation from host trees to the ectomycorrhizal root tips and mycelia. Ashkannejhad and Horton^42^ showed that ectomycorrhizal symbiosis functions as a critical factor for pine establishment in the coastal dune ecosystem in Oregon. In our study, fungal abundance and the fungal/bacterial ratio were highest in the 0–10 cm layer in plot 4 (Fig. 8b,c), near a pine forest. Although it is not possible to separately evaluate the contributions of root respiration and mycorrhizal CO_2_ efflux to *R*_s_ in our study, this finding implies a possible contribution of ectomycorrhizal root tips and mycelial networks to *R*_s_. The *R*_a_/*R*_s_ to a depth of 50 cm was 0.62 in plot 4, and some fraction of the contribution would be caused by ectomycorrhizal CO_2_ efflux.

SOC is the source of *R*_h_ and also strongly influences *R*_s_. For example, a study by Li et al.^43^ in an alpine meadow ecosystem on the Qinghai-Tibetan Plateau indicated positive relationships between SOC and *R*_s_ and their components (*R*_h_ and *R*_a_). Morisada et al.^44^ estimated the average SOC stock in Japanese forests to a depth of 30 cm as 9.0 kg C m^−2^. Compared with that amount, the SOC stock at our study site (maximum 0.3 kg C m^−2^ in plot 4) was remarkably limited. Therefore, the relatively small contribution of *R*_h_ to *R*_s_ is theoretically possible under the SOC-limited condition of our study site. Our microbial analyses revealed a variety of bacterial and fungal abundance in each plot (Fig. 8), suggesting that the contribution of *R*_h_ to *R*_s_ varies among plots. Our result indicates that the zonal distribution of plant species in a coastal dune ecosystem significantly influenced the abundance of the soil microbiota. In addition, the significantly greater abundance of microbiota and SOC in the shallower layers (0–10 and 10–20 cm) compared with the deeper layer (20–30 cm) in plot 4 suggest that the shallower layer is a larger source of *R*_h_ compared with the deeper layer.

There are uncertainties regarding our findings on the influence of BPB on *R*_s_. First, trench treatment in our study was limited to the depth of 50 cm, and we could not exclude the influence of roots in the deeper layer. The influence was likely relatively minor in plots 3 and 4 because more than 90% of BPB was concentrated in the upper 50 cm in those plots (Fig. 5). In plots 1 and 2, however, BPB was distributed at depths greater than 50 cm (Fig. 5), which certainly caused underestimation of the contribution of *R*_a_ to *R*_s_ in plots 1 and 2. We have limited information regarding the magnitude of the *R*_a_/*R*_s_ in deeper layers as compared with that in shallower layers. Pregitzer et al.^45^ reported that root respiration in the surface layer (0–10 cm) was up to 40% higher than that at 20- to 30-cm and 40- to 50-cm depth in sugar maple forests in Michigan. According to the report, it is possible that *R*_a_ in the deeper layer below 50 cm less contributed to *R*_s_ compared with the *R*_a_ in the shallower layer (0–50 cm) in our study. Even though, there is no report showing the influence of deeper roots below 50 cm on *R*_s_ compared with the roots in the shallower layer. Therefore, the uncertainty of the *R*_a_/*R*_s_ in plots 1 and 2 is larger than that in plots 3 and 4.

Second, there is uncertainty about the influence of dead root decomposition and disturbance by trench treatment on *R*_s_ in trenched plots, and this may also have caused an underestimation of the *R*_a_/*R*_s_. Carbon input as dead roots to trenched plots is inevitable in the trench treatment^6^. Some studies applied a correction for *R*_a_/*R*_s_ by conducting root bag experiments^46,47^, whereas others did not^23,48^. In our study, we did not apply any correction for the influence of BPB on *R*_s_ because of the short experimental period (we collected root samples to a depth of 50 cm in all subplots within 2 months after trench treatment). Previous studies reported that the influence of disturbance accompanied by trench treatment ceased several months after the treatment^47,49^. For example, Lee et al.^47^ reported that *F*_c_ of trenched plots was higher compared with that of control plots until 1–2 months after trench treatment, and *F*_c_ in trenched plots significantly decreased after that period. In our study, the significant decrease of *F*_c_ in trenched plots compared with control plots was confirmed 15 days after trench treatment. It appears that the early occurrence of a *F*_c_ decrease after trench treatment was due to the relatively low soil temperature in November (Fig. 1a), such that the influence of dead root decomposition on *F*_c_ might be minor.

Third, seasonal dynamics of the *R*_a_/*R*_s_ were not considered in this study. Previous studies reported that the *R*_a_/*R*_s_ in the growing season was larger than that in the dormant season^50–52^. However, Lee et al.^47^ observed an exceptionally large contribution of *R*_a_ to *R*_s_ in November (71%) in a cool-temperate deciduous forest in central Japan. Our measurement to assess the *R*_a_/*R*_s_ was conducted in November, the transitional period from the growing season to the dormant season. Because we did not consider seasonal trends in our estimation of the *R*_a_/*R*_s_ in a coastal dune ecosystem, this may have led to under-or overestimation.

## Conclusion

The dynamics of *R*_s_ were greatly influenced by abiotic and biotic factors in a coastal dune ecosystem. Our findings demonstrated that seasonal dynamics of *R*_s_ were controlled by soil temperature, but drought stress also strongly influenced *R*_s_ in the dry summer period, and the response of *R*_s_ to drought varied among plots dominated by different vegetation species. In addition, *R*_a_ made a large contribution to *R*_s_, and the distribution of BPB appeared to be a factor controlling the spatial dynamics of *R*_s_. Furthermore, the microbial analysis suggested that the zonal distribution of vegetation in the dunes also strongly influenced microbial abundance, indicating that the contribution of the microbial community to *R*_s_ likely differs among measurement plots. These findings support our hypothesis that relationships between the abiotic and biotic controlling factors and *R*_s_ vary among plots dominated by different vegetation species, reflecting the zonal vegetation distribution patterns in a coastal dune ecosystem. Our study provides important data for further examination of coastal dune ecosystems from the viewpoint of carbon cycle analysis.

## Materials and methods

### Site description

The study site (about 1 ha) is within a coastal dune ecosystem (35° 32′ 26.0″ N, 134° 12′ 27.5″ E) located at the Arid Land Research Center of Tottori University, Tottori, Japan. The mean annual temperature is 15.2 °C, and the mean total precipitation is 1931 mm, based on records collected from 1991 to 2020 at the Tottori observation station of the Japan Meteorological Agency. Dominant plant species around the measurement plot were *Vitex rotundifolia* and *Artemisia capillaris*. *Carex kobomugi* and *Ischaemum anthephoroides* were also scattered around the coastal side of the study site, and planted *Pinus thunbergii* trees cover the inland side.

### Experimental design

**Figure 9:**
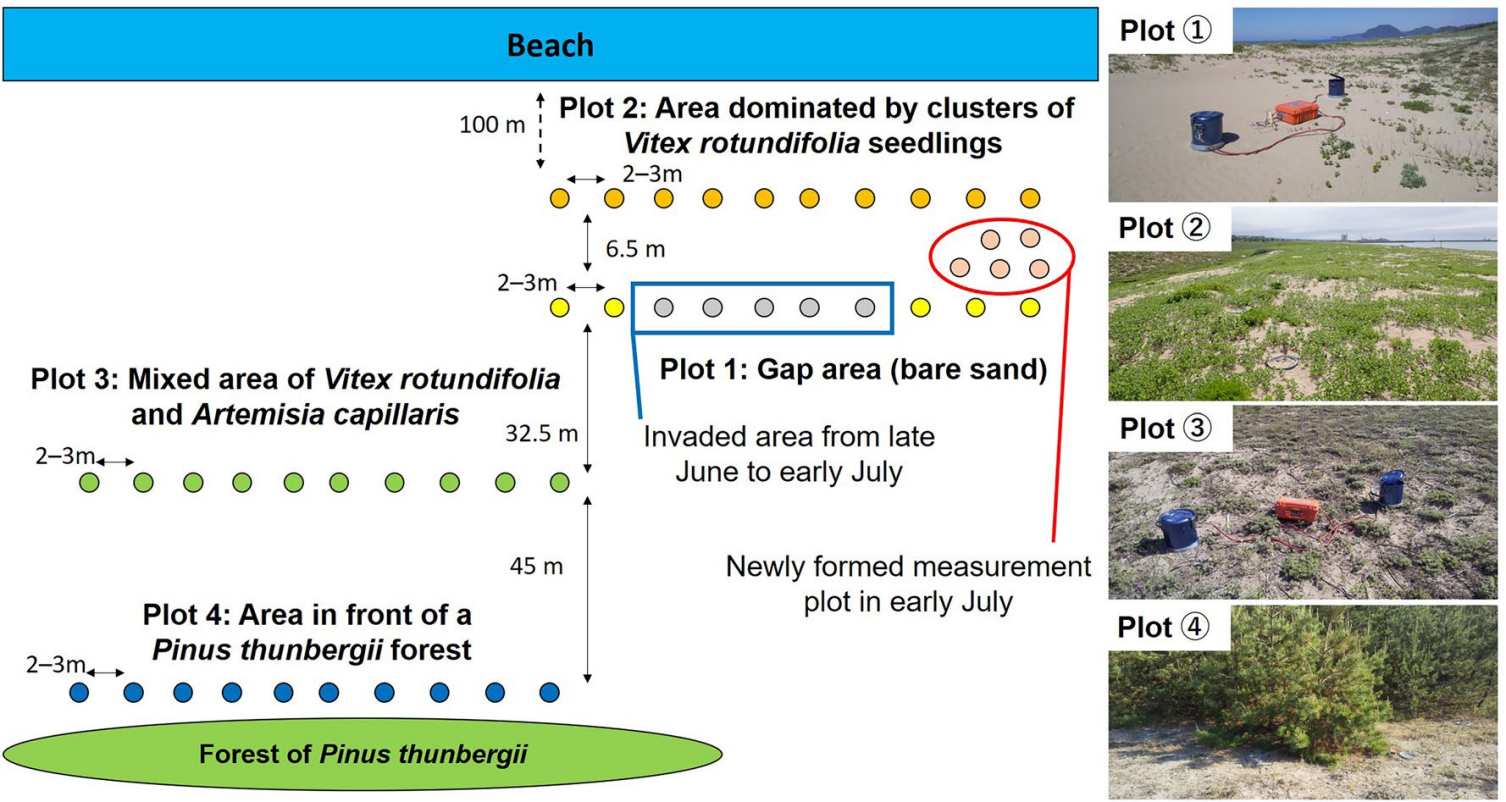
Diagram and photos of measurement plots in the focal coastal dune ecosystem. *Vitex rotundifolia* and *C*. *kobomugi* invaded a part of plot 1 in late June to early July, after the first *R*_s_ measurement on 15 June. Therefore, we set new measurement points for plot 1 in early July.

In May 2020, we established four measurement plots at the study site (Fig. 9). Plot 1 was a gap area surrounded by *V. rotundifolia* seedlings. Plot 2 consisted of clusters of *V. rotundifolia* seedlings and was adjacent to plot 1. Within plots 1 and 2, *C. kobomugi* and *I. anthephoroides* were also scattered. Plot 3 was in a mixed area of *V. rotundifolia* and *A. capillaris*; this plot was in the center of the study site. Plot 4 was located in front of *P. thunbergii* trees and was in the most inland area of the study site. On 10 June 2020, we set an environmental measurement system at the center of the study site adjacent to plot 3, and we then obtained continuous data for soil temperature and soil moisture. In each plot (main plot), we set 10 plastic (polypropylene) collars (*n* = 10) before the start of the *R*_s_ measurement. We measured *R*_s_ every 2 weeks from 15 June to 2 December 2020 in the main plots. *Vitex rotundifolia* and *C. kobomugi* invaded a part of plot 1 in late June and early July, after the first *R*_s_ measurement on 15 June. Therefore, we set new measurement points for plot 1 in early July (Fig. 9), and flux calculations for plot 1 were conducted after removing data from the invaded area measured on June 15.

### Environmental measurement system

The environmental measurement system was composed of a data logger (CR1000, Campbell Scientific Inc., Logan, UT, USA), battery (SC dry battery, Kind Techno Structure Co. Ltd, Saitama, Japan), solar panel (RNG-50D-SS, RENOGY International Inc., Ontario, CA, USA), charge controller (Solar Amp mini, CSA-MN05-8, DENRYO, Tokyo, Japan), thermocouples (E type), and soil moisture sensors (CS616, Campbell Scientific Inc.). The data logger, battery, and charge controller were kept in a plastic box to avoid exposure to rainfall and sand. Each end of the thermocouple was inserted into a copper tube (4-mm inner diameter, 5-cm length) and affixed with glue. To measure the reference soil temperature at different depths, copper tubes enclosing E-type thermocouples were buried horizontally in the sand at depths of 5, 10, 30, and 50 cm (*n* = 1 for each depth) at the center of plot 3 as reference soil temperature (the data was recorded every 30 min). In addition, we set stand-alone soil temperature sensors (Thermochron SL type, KN Laboratories, Inc. Osaka, Japan) at the center of plots 1 and 4 at depths of 5, 10, and 30 cm (*n* = 1 for each plot, each depth), and they recorded soil temperature data every 30 min. Reference soil temperature at the depth of 5, 10, and 30 cm was used for gap-filling for soil temperature measured by stand-alone sensors at each depth and plot. Soil moisture sensors were buried horizontally in the sand at a depth of 30 cm in the center of plots 1, 3, and 4 (*n* = 1 for each plot) and recorded data every 30 min. Raw values of soil moisture sensors were converted to volumetric soil moisture (%) using a calibration line from 0 to 15% measured in the laboratory using dune sand and three sensors (CS616) referring to the procedure of Bongiovanni et al.^53^. Data for precipitation at the local meteorological observatory in Tottori was downloaded from the home page of the Japan Meteorological Agency (https://www.data.jma.go.jp/gmd/risk/obsdl/index.php).

### ***R***_s_ measurement in the main plots

Polypropylene collars (30-cm inner diameter, 5-cm depth, *n* = 10) were set in each measurement plot in late May 2020. The first *R*_s_ measurement was conducted on 15 June 2020. However, *V. rotundifolia* and *C. kobomugi* then invaded about half of the gap area of plot 1, so on 1 July we set 5 new polypropylene collars for plot 1 to replace the 5 invaded measurement points (Fig. 9). The second *R*_s_ measurement was conducted on 2 July, and all polypropylene collars then remained in the same position until the end of the measurement period.

*R*_s_ was measured using an automated closed dynamic chamber system^54^ composed of two cylindrical aluminum chambers (30 cm diameter, 30 cm height) equipped with thermistor temperature sensors (44006, Omega Engineering, Stanford, CA, USA) for measuring air temperature inside the chamber during *R*_s_ measurement. Those chambers were connected to a control box equipped with a pump, data logger (CR1000, Campbell Scientific Inc.), CO_2_ analyzer (Gascard NG infrared gas sensor, Edinburgh Sensors, Lancashire, UK), and thermometer (MHP, Omega Engineering). The composition of the control box is basically the same as used in previous studies^54,55^. The measurement period for each point was 3 min, and the CO_2_ concentration and air temperature inside the chamber were recorded every 5 s. During the measurement, another chamber was set on the next polypropylene collar with the lid opened, and the next measurement was started at that moment of finishing the previous measurement by automatically closing the chamber lid on the next polypropylene collar in the same plot. Soil temperature at a depth of 0–5 cm was recorded simultaneously by inserting the rod of the thermometer vertically into the soil surface near the polypropylene collar (about 1–2 m from the collar).

*R*_s_ was calculated by using the following equation:

1 $$R_{{\text{s}}} = \frac{{PV}}{{RS(T_{{{\text{air}}}} + 273.15)}}\frac{{\partial C}}{{\partial t}},$$

where *P* is the air pressure (Pa), *V* is the effective chamber volume (m^3^), *R* is the ideal gas constant (8.314 Pa m^3^ K^−1^ mol^−1^), *S* is the soil surface area (m^2^), *T*_air_ is the air temperature inside the chamber (°C). ∂C/∂t is the rate of change of the CO_2_ mole fraction (μmol mol^−1^ s^−1^), which was calculated using least-squares regression of the CO_2_ changes inside the chamber^12^. For the flux calculation, we removed data for the first 35 s (dead band) of each measurement as an outlier.

### Trench treatment and soil CO_2_ efflux (***F***_c_) measurement in subplots

In November 2020, we conducted root-cut treatment (trench treatment) in subplots using polyvinyl chloride (PVC) tubes to estimate the contribution of *R*_a_ to *R*_s_ in the soil layer above 50 cm in each plot (*R*_a_50_/*R*_s_). Small PVC collars (10.7 cm inner diameter, 5 cm depth, *n* = 10 for each plot), with the upper ends about 1–2 cm above the soil surface, were set in subplots adjacent to the main plots on 23 October 2020. *R*_s_ was measured in subplots using two cylindrical mini PVC chambers (11.8 cm inner diameter at the bottom, 30 cm height, equipped with the same thermistors as cylindrical aluminum chambers for air temperature measurement) connected to the same control box as used for *R*_s_ measurement in the main plots. The measurement period was 3 min, and the measurement procedure and the flux calculation were the same as the main plot. *R*_s_ was first measured in subplots on 3 November to examine the spatial variation of *R*_s_ before trench treatment. Using the data, we selected subplots to conduct trench treatment and control plots for comparison, while aiming to achieve a minimal difference in the average *R*_s_ between control and pre-trenched plots. On 4 November, we inserted PVC tubes (10.7 cm inner diameter, 50 cm length) into about half (*n* = 3–5) of the subplots (the same position as PVC collars were set on 23 October) by using a hammer and aluminum lid until the upper end of each PVC tube was 1–2 cm above the soil surface to exclude roots to a depth of about 50 cm. On 19 November, after 15 days of trench treatment, respiration was measured in the same subplots.

The *R*_a_50_/*R*_s_ was calculated as follows:

2 $$R_{{{\text{a}}\_{5}0}} /R_{{\text{s}}} = (F_{{{\text{c}}\_{\text{control}}}} -F_{{{\text{c}}\_{\text{trenched}}}}) /F_{{{\text{c}}\_{\text{control}}}} ,$$

where *F*_c_trenched_ and *F*_c_control_ (= *R*_s_) are the *F*_c_ values in trenched and control plots on 19 November, respectively.

In late December 2020, all the belowground plant biomass (BPB) in subplots (control and trenched plots) to a depth of 50 cm was collected for biomass analysis, about 2 months after trench treatment. In the laboratory, all the collected plant materials were washed and oven-dried for 72 h at 70 °C, and then the dry weight of the BPB samples was measured.

### Biomass measurement

We conducted BPB analysis from 18 May to 8 June 2021 in each plot (*n* = 1). At that time, 100 cm × 100 cm sampling plots near the CO_2_ measurement plots (100 cm × 100 cm for plots 2–4 and 50 cm × 50 cm in plot 1 because of the narrow gap area) were dug to a depth of 100–220 cm, according to the root distribution in each plot, and all plant materials were collected by passing the soil through 5- to 7-mm sieves. Once we reached a depth where no roots were visible, no more digging was conducted. In plots 2 and 3, stolons of *V. rotundifolia* were difficult to distinguish from roots if underground. Therefore, we defined plant material as BPB if it was underground. In the laboratory, all of the collected plant materials were washed and air-dried at room temperature for 0–6 days depending on the biomass. After that, samples were oven-dried for 15–25 h at 70–80 °C, and the dry weight of those samples was then measured.

### Soil organic carbon and nitrogen

On 21 October 2020, soil pits were dug to a depth of 50 cm near each plot (*n* = 3), and soil core samples were collected. Cylindrical stainless core samplers (5 cm diameter, 5 cm height, 100 cc) were horizontally inserted into the soil pit at depths of 0–5, 5–10, 10–20, and 20–30 cm. In the laboratory, soil core samples were weighed and oven-dried at 105 °C for 48 h, and the dry weight was measured. Oven-dried soil samples were sieved with a 2-mm-pore stainless wire mesh screen, and visible fungal mycelia in soil samples from plot 4 were removed as well as possible. Sieved samples were ground with an agate mortar. Samples (fine powder) were oven-dried for 24 h at 105 °C and weighed before SOC and nitrogen analysis. About 1.5 g of powdered samples were used for the analysis. Organic carbon content (combustion at 400 °C) and total nitrogen in samples were analyzed using a Soli TOC cube (Elementar Analysensysteme GmbH, Langenselbold, Germany) by the combustion method.

### Microbial abundance

On 21 October 2020, soil samples for microbial analysis were collected at the same time as soil core sampling for SOC and nitrogen analysis. Soil samples were collected at depths of 0–10, 10–20, and 20–30 cm using a stainless spatula and placed individually in a polyethylene bag. The bags were kept in a cooler box with ice in the field and then placed in a freezer (− 30 °C) in the laboratory soon after sampling.

DNA was extracted from 0.5 g of the fresh soils using NucleoSpin Soil (Takara Bio, Inc., Shiga, Japan) according to the manufacturer’s instructions (SL1 buffer), and the extracts were stored at − 20 °C until further analysis. Bacterial and archaeal 16S rRNA and fungal internal transcribed spacer (ITS) gene were targeted to investigate the microbial abundance. Bacterial and archaeal 16S rRNA (V4 region) and fungal ITS were determined using the universal primer sets 515F/806R and ITS1F_KYO2/ITS2_KYO2, respectively^56,57^.

For qPCR, samples were prepared with 10 μL of the KAPA SYBR Fast qPCR kit (Kapa Biosystems, Wilmington, MA, USA), 0.8 μL of forward primer, 0.8 μL of reverse primer, and 3 μL of 1–50 × diluted soil DNA. Nuclease-free water was added to make up to a final volume of 20 μL. Cycling conditions of 16S rRNA were 95 °C for 30 s, followed by 40 cycles at 95 °C for 30 s, 58 °C for 30 s, and 72 °C for 1 min. Cycling conditions of ITS were 95 °C for 30 s, followed by 40 cycles at 95 °C for 30 s, 55 °C for 1 min, and 72 °C for 1 min. A melting curve analysis was performed in a final cycle of 95 °C for 15 s, 60 °C for 1 min, and 95 °C for 15 s. High amplification efficiencies of 99% for bacterial and archaeal 16S rRNA genes and 101% for the fungal ITS were obtained based on the standard curves.

### Data analysis

To examine the environmental response (soil temperature and soil moisture) of *R*_s_, nonlinear and quadratic regression models were applied. We conducted *F*-tests by comparing the regression model to a constant model whose value is the mean of the observations (significance set at *p* < 0.05). For the temperature response analysis of *R*_s_, we used the following equation^58^:

3 $$R_{{\text{s}}} = R_{{{\text{ref}}}} {\text{e}}^{{E_{0} \times \left( {\frac{1}{{T_{{{\text{ref}}}} - T_{0} }} - \frac{1}{{T_{{\text{s}}} - T_{0} }}} \right)}} ,$$

where *R*_ref_ (µmol CO_2_ m^−2^ s^−1^) is the CO_2_ efflux at a specified reference soil temperature (*T*_ref_: 283.15 K), *E*_0_ is a fitting parameter, *T*_0_ is the soil temperature when *R*_s_ is zero (227.13 K), and *T*_s_ is the observed soil temperature (K) at different depths (0–5, 5, 10, 30, 50 cm). Based on the 1-year soil moisture data between 11 June 2020 and 10 June 2021, we defined the period when soil moisture was below the annual average − 2SD (= 3.9%) as a drought period (10 August to 4 September), and we conducted nonlinear regression for the temperature response of *R*_s_ with and without *R*_s_ data during the drought period. To avoid the confounding effects of soil temperature and soil moisture, we first divided the observed value by the simulated value of *R*_s_ based on the temperature response curve (the curve was calculated without data collected in late August 2020, during a drought period). The temperature-normalized *R*_s_, *R*_sN_, was used to analyze the relationship between soil moisture and *R*_s_^59^. The relationship was fitted with the following quadratic regression:

4 $$R_{{{\text{sN}}}} = c_{1} {\uptheta }^{2} + c_{2} {{\uptheta + }}c_{3} (c_{1} < 0),$$

where θ is the volumetric soil moisture (%) and *c*_1_, *c*_2_, and *c*_3_ are fitting parameters.

To examine the relationship between BPB and *R*_s_, we referred to the logarithmic relationship between root biomass and *R*_s_ in a previous study in a forest ecosystem^40^, and we calculated the natural logarithm of BPB to a depth of 50 cm (ln BPB (g)). Correlation analysis (Spearman’s rank correlation, significance set at *p* < 0.05) between the ln BPB in each subplot and *R*_s_ was conducted.

To examine the relationship between SOC stock (g C m^−2^) and microbial abundance (log copies of genes g^−1^ soil), linear regression analysis and *F*-tests were performed (significance set at *p* < 0.05).

We performed all the above-mentioned statistical analyses using Sigmaplot 14.5 software (Systat Software, San Jose, CA, USA, https://systatsoftware.com/sigmaplot/).

The soil microbial abundance was assessed by using two-way analysis of variance (ANOVA) in R 4.0.3., and then Tukey’s test was performed to analyze significant differences between each treatment. Differences were considered statistically significant at *p* < 0.05 (two-sided test).

### Ethics statement

The collection of plant materials in this study complied with relevant institutional, national, and international guidelines and legislation.

## Data Availability

The datasets generated in this study are available from the corresponding author according to reasonable requests from readers.
